# Construction of a Three-Dimensional Calcific Aortic Valve Disease Model Using Human iPSC-Derived Valvular Interstitial Cells

**DOI:** 10.1007/s12015-025-11030-3

**Published:** 2025-12-04

**Authors:** Ruikang Guo, Zhen Qi, Ping Qiu, Dogukan Mizrak, Bo Yang

**Affiliations:** 1 Department of Cardiac Surgery, University of Michigan, Ann Arbor, MI 48109 USA; 2 Department of Cardiac Surgery, Second Xiangya Hospital, Central South University, Changsha, 410011 P. R. China

**Keywords:** Calcific aortic valve disease, Valvular interstitial cells, Induced pluripotent stem cells, 3D tissue construct, FOXO1, Metformin

## Abstract

Calcific aortic valve disease (CAVD) is a progressive and life-threatening condition characterized by fibrocalcific remodeling of the valve leaflets. Valvular interstitial cells (VICs) are central mediators of calcific aortic valve disease (CAVD), as their osteogenic trans-differentiation drives pathological matrix remodeling and calcium deposition within the valve leaflets. Human-induced pluripotent stem cell-derived valvular interstitial cells (hiVICs) represent a promising patient-specific platform for disease modeling. While primary VICs (pVICs) readily undergo mineralization under osteogenic stimulation, hiVICs fail to calcify in conventional two-dimensional (2D) cultures. Our data suggests that the inability of hiVICs to calcify in 2D culture is related to FOXO1 (Forkhead box protein O1) activity, which suppresses the osteogenic transcriptional program by inhibiting RUNX2 (Runt-related transcription factor 2). To address this limitation, we then developed a three-dimensional tissue ring construct using hiVICs. When cultured in osteogenic medium, these constructs exhibited robust calcification, as confirmed by Alizarin Red and Von Kossa staining. FOXO1 was also identified as a mediator of calcification in the tissue ring constructs. Metformin treatment restored *FOXO1* expression and inhibited calcification, while AS1842856, a selective FOXO1 inhibitor, exacerbated tissue construct mineralization and led to a near-complete tissue collapse. In summary, we establish a functional 3D hiVIC-based model of CAVD enabling mechanistic investigation and pharmacological screening and identify FOXO1 as a critical regulator of osteogenic transition.

**Graphical Abstract:**

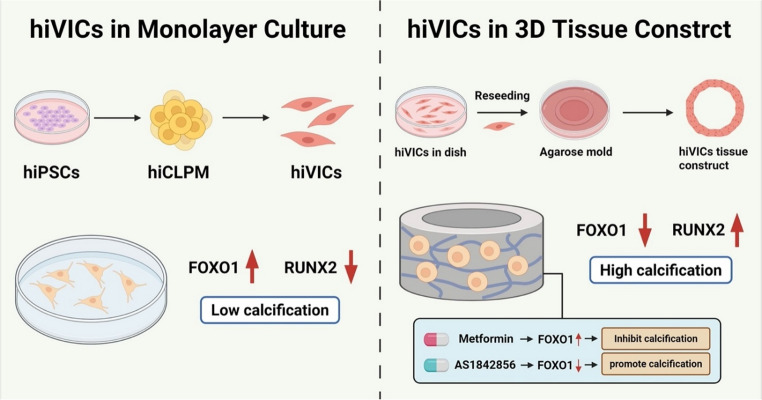

**Supplementary Information:**

The online version contains supplementary material available at 10.1007/s12015-025-11030-3.

## Introduction

 Calcific aortic valve disease (CAVD) is a progressive disorder characterized by the gradual thickening, stiffening, and calcification of the aortic valve leaflets [1]. It represents the most prevalent form of valvular heart disease in the aging population and is associated with significant morbidity and mortality [2]. Despite the advances in diagnostic imaging and interventional cardiology, there are currently no approved pharmacological therapies to manage CAVD. Surgical aortic valve replacement (SAVR) and transcatheter aortic valve replacement (TAVR) remain as mainstay treatments [3]; however, both approaches carry considerable risks, particularly in elderly and high-risk patients [4]. This underscores the need to understand the pathogenesis of CAVD [5] for alternative, non-invasive therapeutic strategies.

Valve interstitial cells (VICs) and valve endothelial cells (VECs) are the two primary cell types comprising normal heart valves, with VICs playing a central role in the formation and progression of CAVD. A critical early event in CAVD progression is the osteogenic differentiation of human valve interstitial cells (hiVICs), which induces profound changes in human aortic valves including increased collagen deposition and formation of nucleation sites for calcium accumulation [6]. Given the central role of hiVICs in initiating and propagating valvular calcification, targeting the mechanisms underlying their osteogenic transition presents a promising avenue for therapeutic intervention [7]. Therefore, identifying molecular pathways and pharmacological agents capable of inhibiting hiVIC osteogenic differentiation is critical for an effective CAVD treatment [8].

In vitro CAVD research heavily relies on the use of primary VICs; however, the limited accessibility, low proliferative capacity, and significant heterogeneity of primary VICs hinder the development of CAVD drugs. As an alternative, an iPSC differentiation protocol for valvular interstitial cells (hiVICs) has recently been developed, providing an unlimited access to VICs [9]. To more accurately recapitulate the complex cellular and extracellular dynamics of CAVD, recent efforts have been directed toward the development of three-dimensional (3D) in vitro valve models. Traditional two-dimensional (2D) culture systems, while useful for mechanistic studies, fail to capture the dynamic cell–matrix interactions present in native valve tissue. In contrast, 3D models provide a more physiologically relevant microenvironment that better mimics the structural and mechanical properties of the aortic valve [10]. These models often incorporate biomimetic scaffolds composed of natural or synthetic hydrogels, decellularized ECM, or bioprinted constructs, which can be seeded with primary VICs [11,12]. Some advanced systems also integrate biomechanical stimuli such as cyclic strain and fluid shear stress to simulate the hemodynamic forces experienced in vivo [13]. Importantly, 3D platforms enable the investigation of spatially regulated calcification processes and allow the screening of therapeutic agents in a more predictive context. As such, 3D valve models represent a significant step forward in bridging the gap between basic research and translational applications in the study of CAVD.

In this study, we developed a 3D tissue ring model using hiPSC-derived hiVICs to investigate the molecular and mechanical drivers of aortic valve calcification, with a specific focus on FOXO1–RUNX2 signaling and its pharmacological modulation. Our self-assembled, scaffold-free 3D tissue construct enables modeling of human CAVD using hiVICs in a physiologically relevant yet technically accessible system.

## Materials and Methods

### Primary Cell Extraction and Culture

As previously reported [14], human aortic valves were used to extract primary human valve interstitial cells (pVICs). Briefly, the aortic valve leaflets were carefully sliced into tiny pieces, digested in 1 mg/mL type I collagenase for 30 min at 37 °C, and then incubated for 12 h at 37 °C in 5% CO2 with a new solution of 2 mg/mL type I collagenase. The pVICs were then resuspended and cultured in high glucose Dulbecco’s modified Eagle’s medium (DMEM, Gibco, Invitrogen, Carlsbad, CA, USA) supplemented with 10% fetal bovine serum (FBS) (Gibco, Invitrogen, Carlsbad, CA, USA) in a humidified atmosphere with 5% CO2 at 37 °C. Next, the cell suspension had been gently spun for 10 min at 1000 rpm to pellet the cells prior to the seeding.

### HiPSCs to hiVICs Differentiation

The hiPSCs to hiVICs differentiation protocol was reported before [9]. The hiPSC generation and maintenance was previously described [15]. Briefly, hiPSCs were plated on Matrigel-coated surfaces in mTeSR1 medium supplemented with Y-27632 (10 µM). After 24 h, differentiation was initiated using a Neurobasal/DMEM-F12-based medium containing B27, N2, CHIR-99,021 (8 µM), and BMP4 (25 ng/mL) to induce cardiac lateral plate mesoderm. On day 3, cells were switched to StemPro-34 SFM supplemented with VEGF-165 (200 ng/mL) and forskolin (1 µM), with daily medium changes. On day 6, cells were dissociated and maintained in DMEM with 10% FBS.

### RT-PCR and Cell Immunofluorescence

Total RNA was isolated using the RNeasy Mini Kit (QIAGEN, Germany) according to the manufacturer’s instructions. Reverse transcription was performed with 1 µg of total RNA using a standard cDNA synthesis protocol. Quantitative real-time PCR was conducted with iTaq™ Universal SYBR^®^ Green Supermix (Bio-Rad, USA) on a CFX96 Touch Real-Time PCR Detection System (Bio-Rad). Primers were designed to span exon–exon boundaries when possible, and GAPDH was used as the internal reference. Relative transcript abundance was calculated by the 2^–ΔΔCt method. Primer sequences are listed in Supplementary Table 1**.** Experiments were performed in three independent biological replicates (*n* = 3).

Cells were grown on sterile glass coverslips in 24-well plates and fixed with 4% paraformaldehyde for 15 min at room temperature. Following permeabilization with 0.1% Triton X-100 for 10 min, samples were blocked in 5% BSA/PBS for 1 h. Coverslips were then incubated overnight at 4 °C with primary antibodies diluted in 1% BSA/PBS. After washing, fluorophore-conjugated secondary antibodies were applied for 1 h at room temperature in the dark. Nuclei were counterstained with DAPI (1 µg/mL) for 5 min, and coverslips were mounted using antifade mounting medium. Images were captured using a fluorescent microscope. Experiments were performed in three independent biological replicates (*n* = 3).

### Calcification Induction and Staining

pVICs and hiVICs were seeded in 12-well plates and cultured to confluence in either high phosphate medium (HP), consisting of DMEM (high glucose) supplemented with 2% FBS,1% penicillin/streptomycin and 0.025 g sodium phosphate, or osteogenic medium (OM), composed of DMEM (high glucose) with 2% FBS, 1% penicillin/streptomycin, 50 µg/mL ascorbic acid, 100 nmol Dexamethasone and 10 mM β-glycerophosphate. After 21 days of induction, cells were fixed with 4% paraformaldehyde for 30 min at room temperature and subsequently washed with ultrapure water. The calcified matrix deposition was assessed by staining with Alizarin Red S (Sigma-Aldrich) and BCIP/NBT liquid substrate system (Sigma-Aldrich) for 30 min, followed by thorough washing with distilled water to remove excess dye. Mineralized nodules were observed under a light microscope. Experiments were performed in three independent biological replicates (*n* = 3).

### Construction of hiVICs Tissue Construct, Calcification Induction, and Drug Treatment

The preparation protocol of the tissue ring model was reported before [16–18]. Briefly, dimethyl siloxane molds were immersed in 70% ethanol for 30 min, air-dried, and exposed to ultraviolet light for 1 h. A 2% agarose gel solution in DMEM was prepared by heating in a water bath for 30 min or microwaving for three 30-second intervals. The gel solution was transferred into the mold, covered with a sterile cover glass, and solidified at room temperature for 30 min. After solidification, the gel was transferred to a six-well plate, covered with DMEM/F12 medium (1% penicillin/streptomycin), and incubated at 37 °C for 24 h.

hiVICs were washed, digested with Trypsin, and centrifuged. The cell suspension was resuspended in 10% FBS solution, counted, and adjusted to 0.2–0.5 × 10⁶ cells per well. The agarose gel in the six-well plate was washed, and 100 µL of cell suspension was added to each well. After a 30-minute incubation at 37 °C for attachment, fresh 10% FBS DMEM medium was added and incubated for another 24 h. The medium was then replaced with a tissue construct culture medium consisting of DMEM (high glucose, 20% FBS, 1 ng/mL TGF-β1, 1% penicillin/streptomycin, 50 µg/mL proline, 50 µg/mL glycine, 20 µg/mL alanine, 3 ng/mL CuSO₄, and 50 µg/mL ascorbic acid). The plate was incubated at 37 °C, with medium changes every two days, and cultured for 7 days.

After 7 days of tissue construct medium treatment, the medium was replaced with tissue construct OM medium, tissue construct HP medium, plus treatment of 2µM Metformin [19–21], and 5µM AS1842856, a previously reported calcification inducer [22].

### Tissue Ring Construct Mechanical Test and Histology

The tissue ring construct mechanical test protocol was performed as previously reported [23]. The thickness of each construct was determined by measuring both lateral surfaces prior to uniaxial tensile testing. Width and length were also recorded. Mechanical characterization was performed using a uniaxial testing apparatus (RSA-G2, TA Instruments). Constructs were mounted on standard thin-film fixtures (TA Instruments) via rectangular loops inserted through each specimen to apply tension. Samples were elongated to failure at a constant crosshead speed of 10 mm/min. During testing, force (N) and strain (%) were continuously recorded. From the resulting data, two parameters were calculated using MATLAB: (1) ultimate strain, defined as the ratio of elongation at maximum load to the initial gauge length (%), and (2) ultimate tensile stress, calculated as the maximum load divided by the cross-sectional area at the time of measurement (MPa = N/mm²). Paraffin-embedded tissue sections (5 μm) were deparaffinized, rehydrated, and subjected to various histological stains. Hematoxylin and eosin (H&E) staining was performed for general morphology. Masson’s Trichrome staining was used to assess collagen deposition, involving iron hematoxylin, Biebrich scarlet-acid fuchsin, phosphomolybdic/phosphotungstic acid, and aniline blue. Mineralization was evaluated using Von Kossa staining (5% silver nitrate under UV light) and Alizarin Red S staining for calcium deposits. All slides were dehydrated, cleared in xylene, and mounted for imaging. For slice immunofluorescence staining, Paraffin-embedded tissue ring sections were deparaffinized, rehydrated, and subjected to antigen retrieval. After blocking, sections were incubated with primary antibodies against RUNX2 and FOXO1 (Cell Signaling Technology) overnight at 4 °C, followed by fluorescent secondary antibodies. Nuclei were counterstained with DAPI, and images were acquired using a fluorescence microscope. Mechanical test experiments were performed in five independent biological replicates (*n* = 5); Histology experiments were performed in three independent biological replicates (*n* = 3).

### Western Blot

Monolayer cultured cells were lysed in RIPA buffer containing protease and phosphatase inhibitor cocktails. Protein concentrations were determined via bicinchoninic acid (BCA) assay. Equivalent amounts of total protein were separated by SDS–PAGE and electro transferred onto PVDF membranes. After blocking with 5% (w/v) non-fat dry milk or bovine serum albumin, membranes were incubated overnight at 4 °C with primary antibodies against GAPDH (#2118), RUNX2 (#8486), AKT (#9272), p-AKT (Ser 473; #4060), FOXO1 (#2880), and phospho-FOXO1 (Ser256; #9461) (all from Cell Signaling Technology); SMURF2 (ABclonal, A2278). Following incubation with horseradish peroxidase–conjugated secondary antibodies for 1 h at ambient temperature, immunoreactive bands were visualized using enhanced chemiluminescence (ECL) and captured with a ChemiDoc imaging system. Band intensities were quantified using ImageJ software. For hiVIC tissue rings, tissue constructs were cultured with or withour osteogenic medium and drug treatments initiated on day 4. The rings were harvested on day 7, frozen in liquid nitrogen, and ground into fine powder using a pre-chilled mortar and pestle. The powder was lysed in RIPA buffer containing protease and phosphatase inhibitors. Western blots were performed on tissue ring protein preparations as described above. Experiments were performed in three independent biological replicates (*n* = 3).

### Statistics

Statistical analyses were performed using IBM SPSS Statistics 23.0. For comparisons between two groups, Student’s t-test was applied to normally distributed data. Normality of data distribution was assessed using the Shapiro–Wilk test. whereas the Mann–Whitney U test was used for non-normally distributed data. One-way ANOVA followed by Tukey’s HSD post hoc test was conducted for multiple group comparisons. A p-value less than 0.05 was considered statistically significant.

## Results

### Generation and Characterization of Human iPSC-Derived Valvular Interstitial Cells (hiVICs)

**Fig. 1:**
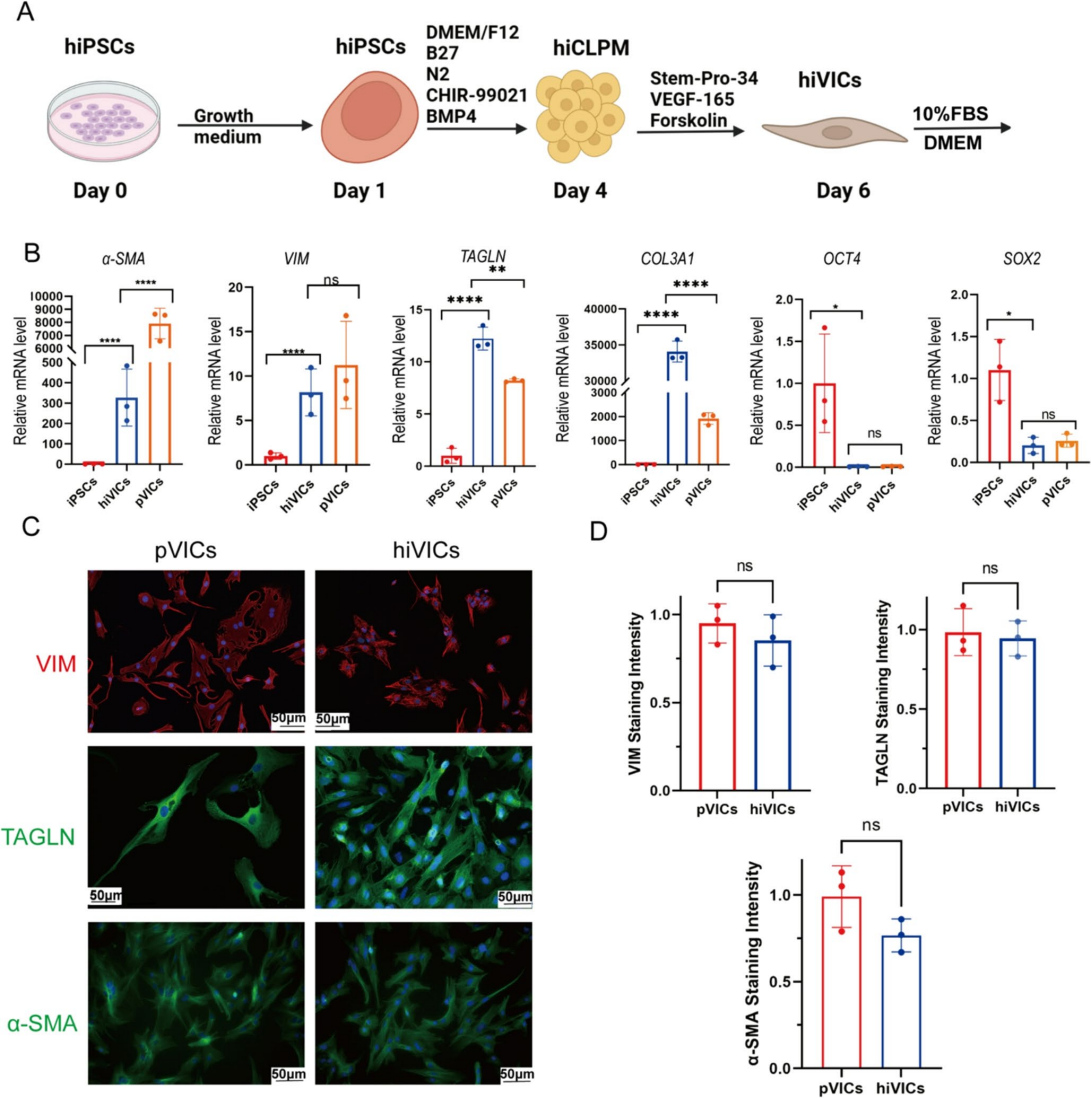
Differentiation and characterization of hiVICs. (**A**) Illustration of differentiation of hiPSCs through the hiCLPM stage to hiVICS. (**B**) Relative expression of VICs markers and stem cell markers in pVICs and hiVICs after differentiation from hiPSCs. *n* = 3 biological replicates. The samples were derived from independent hiVIC and pVIC cell lines. Statistical analysis was performed using one-way ANOVA followed by Tukey’s post hoc test. (**C**) Immunofluorescence staining for interstitial cell markers in hiVICs and pVICS. (**D**) Quantification of mean fluorescence density. *n* = 3 biological replicates. Statistical analysis was performed using the Mann–Whitney U test. Asterisks indicate statistical significance: *P* < 0.05 (*), *P* < 0.01 (**), *P* < 0.001 (***), and *P* < 0.0001 (****)

To establish an in vitro source of human valvular interstitial cells (VICs), we employed a previously described [9] differentiation protocol via a cardiac lateral plate mesoderm (hiCLPM) intermediate stage (Fig. 1A). Given that this procedure results in a mixed population of valvular endothelial cells and valvular interstitial cells, FBS was used to induce the endothelial-to-mesenchymal transition of valvular cells for an additional three-day culture period. Following hiVIC differentiation, we assessed the expression of lineage- and pluripotency-associated markers by quantitative RT-PCR. The mRNA levels of VIC markers, including *α-SMA* (*α-smooth muscle actin*,* ACTA2*), *TAGLN (Transgelin*,* SM22α*), *COL3A1* (*Collagen Type III Alpha 1 Chain*) and *VIM* (*Vimentin)* were significantly elevated in hiVICs compared to undifferentiated hiPSCs, indicating successful mesenchymal induction (Fig. 1B). In addition, expression of the pluripotency genes *SOX2* and *OCT4* was markedly reduced, confirming loss of stemness. When compared to primary VICs (pVICs), hiVICs exhibited moderately lower expression levels of both *ACTA2* and *VIM*, suggesting incomplete maturation toward the native VIC phenotype. To further validate the cellular identity at the protein level, we performed immunofluorescence staining for VIC markers α-SMA, VIM and TAGLN (transgelin/SM22α). Both hiVICs and pVICs exhibited clear cytoplasmic staining for these markers, with cells displaying a characteristic elongated, spindle-shaped morphology (Fig. 1C). Quantitative analysis of mean fluorescence intensity revealed comparable protein expressions of VIM, α-SMA and TAGLN between hiVICs and pVICs (Fig. 1D). Together, these results confirm that hiPSCs can be efficiently differentiated into VIC-like cells, which downregulate stemness programs and acquire basic valvular interstitial cell features albeit remain at a phenotypically intermediate state.

### Monolayer hiVICs are Less Prone to Calcification After Osteogenic Media Stimulation

**Fig. 2:**
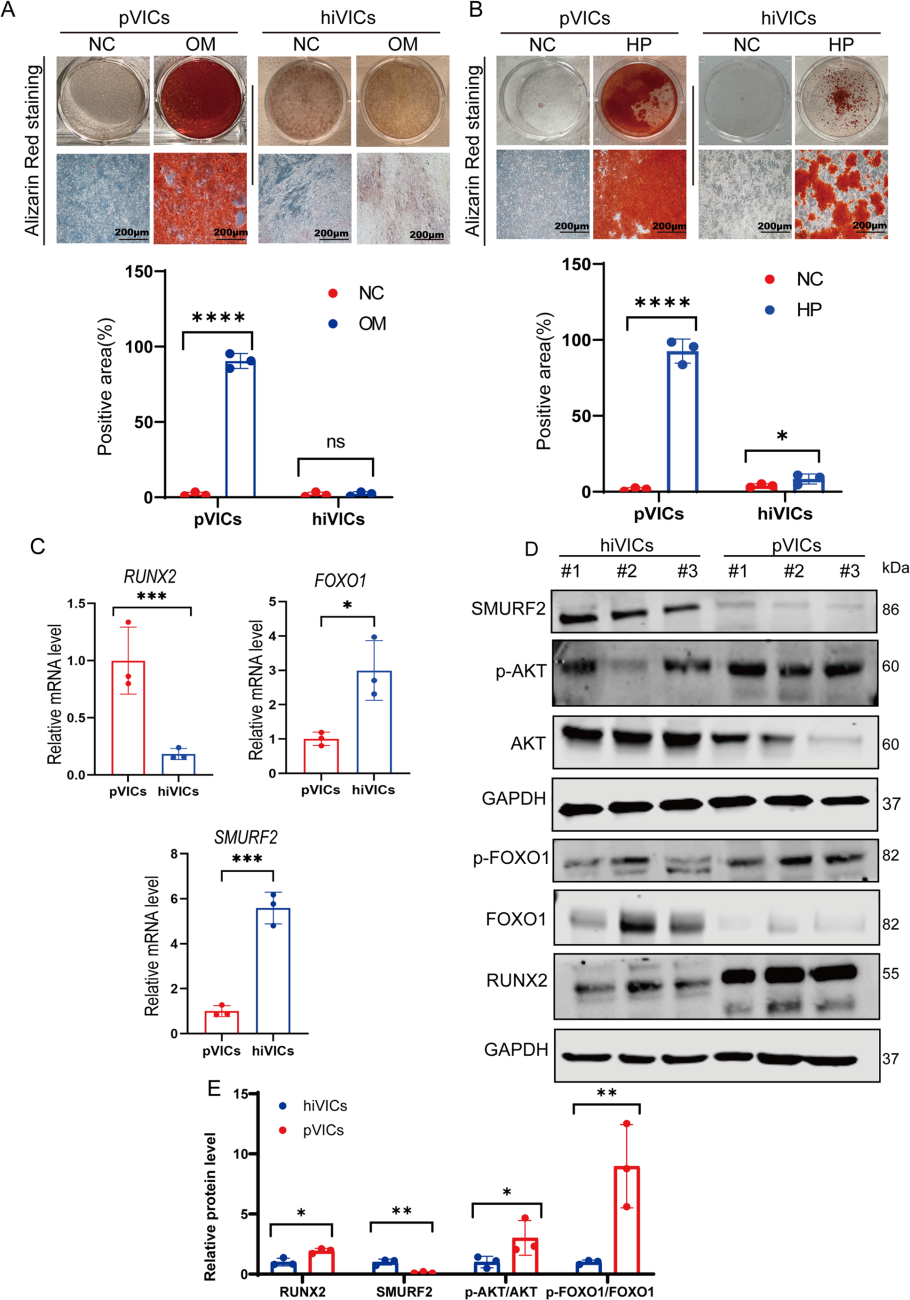
hiVICs are less prone to calcification in 2D culture with OM treatment. (**A**)Alizarin Red Staining of hiVICs and pVICs under OM treatment and their quantification. *n* = 3 biological replicates. (**B**) Alizarin Red Staining of hiVICs and pVICs under HP treatment and their quantification. *n* = 3 biological replicates. (**C**) Relative mRNA expression of *RUNX2*, *SMURF2* and *FOXO1* in pVICs and hiVICs. (**D**-**E**) Relative protein levels of RUNX2, SMURF2, p-AKT/AKT and p-FOXO1/FOXO1 in pVICs and hiVICs. *n* = 3 biological replicates. Statistical significance was determined using Student’s t-test for all comparisons. Asterisks indicate statistical significance: *P* < 0.05 (*), *P* < 0.01 (**), *P* < 0.001 (***), and *P* < 0.0001 (****)

To evaluate the osteogenic potential of hiVICs in two-dimensional (2D) culture, we treated both hiVICs and primary VICs (pVICs) with either osteogenic medium (OM) or high-phosphate (HP) medium. When cultured in OM for 21 days, pVICs exhibited extensive calcium deposition, whereas hiVICs did not show robust mineralization (Fig. 2A). In contrast, HP treatment induced significant calcification in both cell types; however, the intensity and extent of Alizarin Red S–positive nodules remained lower in hiVICs compared with pVICs (Fig. 2B). To understand the molecular basis of this disparity, we examined the expression of key transcriptional regulators of calcification. Previous studies demonstrated that AKT/FOXO1/SMURF2 axis can regulate RUNX2 stability and transcriptional activity, we therefore examined whether they also serve as upstream regulators of RUNX2 changes in hiVICs [22]. Quantitative RT-PCR revealed that hiVICs express significantly lower levels of RUNX2 and higher levels of FOXO1 and SMURF2 mRNA compared to pVICs (Fig. 2C). Western blot confirmed decreased RUNX2 and elevated FOXO1 and SMURF2 in hiVICs (Fig. 2D-E). Notably, phosphorylated AKT (p-AKT), the active form of AKT, and phosphorylated FOXO1 (p-FOXO1), the inactive form of FOXO1, was increased in pVICs, whereas total FOXO1 and AKT were elevated in hiVICs.

Taken together, these data indicate that our hiVICs, unlike pVICs, are intrinsically resistant to OM stimulation in 2D culture, likely due to FOXO1 activity suppressing RUNX2 signaling. The ability of HP to induce partial calcification in hiVICs may be attributed to its capacity to stimulate both RUNX2-dependent and alternative pro-mineralization pathways. Importantly, previous studies [24] have established that osteogenic-medium (OM)–induced calcification is highly dependent on robust activation of the RUNX2-driven osteogenic program, whereas high-phosphate (HP)–induced calcification can also activate RUNX2 while engaging RUNX2-independent pathways, such as phosphate transporter–mediated signaling and apoptosis-associated mineral nucleation.

### HiVICs Acquire Calcification Potential in a 3D Self-Assembled Tissue Construct Under Osteogenic Induction

**Fig. 3:**
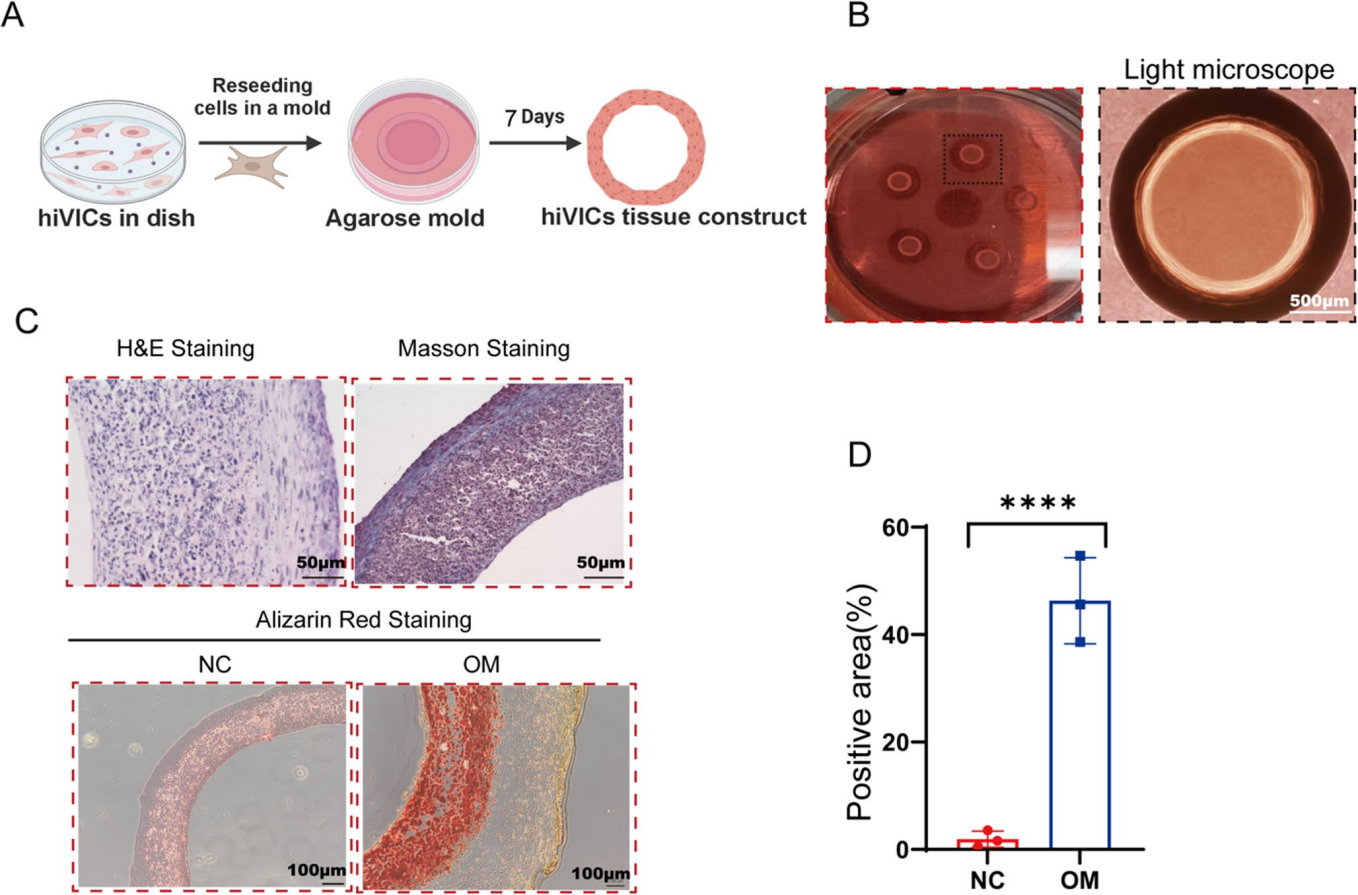
hiVICs can calcify in 3D tissue construct with OM treatment. (**A**) Illustration of hiVIC tissue construct. (**B**)General photographs and light microscope view of hiVICs tissue construct. (**C**)Hematoxylin-Eosin staining, Masson Trichrome, and Alizarin Red staining of hiVICs tissue constructs with or without OM stimulation. (**D**) Quantification of Alizarin Red Staining. *n* = 3 biological replicates. Statistical significance was determined using Student’s t-test. Asterisks indicate statistical significance: *P* < 0.05 (*), *P* < 0.01 (**), *P* < 0.001 (***), and *P* < 0.0001 (****)

Given the inability of hiVICs to calcify under osteogenic stimulation in 2D monolayer cultures, we next tested whether a three-dimensional (3D) microenvironment could promote osteogenic differentiation and mineralization. We employed a tissue ring approach based on cellular self-assembly [16–18](Figure 3A). Agarose provides a biologically inert, non-adherent, and non-degradable environment within this system, enabling cells to spontaneously aggregate and secrete their own ECM without interference from exogenous matrices. This process facilitates the formation of tension-driven, self-assembled ring-shaped structures.

The hiVIC tissue ring constructs demonstrated stable geometry, with macroscopic construct formation visible by eye and under light microscopy (Fig. 3B). Histological analysis using hematoxylin-eosin (H&E) and Masson’s trichrome staining revealed dense cellularity and abundant collagen deposition, confirming that the hiVICs in the 3D configuration actively contributed to ECM remodeling (Fig. 3C). Importantly, when these constructs were cultured in osteogenic medium (OM) for 21 days, clear Alizarin Red-positive staining was observed, indicating significant calcium accumulation within the tissue matrix (Fig. 3C-D). In contrast, constructs cultured in normal control (NC) medium showed no evidence of mineral deposition. These results demonstrate that hiVICs, while resistant to calcification in 2D, can acquire a calcific phenotype when embedded within a 3D tissue construct under osteogenic stimulation. Together, these findings suggest the essential role of the microenvironment in modulating the pathological potential of hiVICs by providing the necessary cues for osteogenic commitment and mineralization, making it a more relevant model for studying early-stage CAVD.

### Pharmacological Modulation Reveals Opposing Effects of Metformin and AS1842856 on Calcification in 3D hiVIC Constructs

To further investigate the role of FOXO1 in modulating calcification, we treated the 3D hiVIC tissue ring constructs with two pharmacological agents: metformin and AS1842856, both reported to regulate FOXO1 signaling. Metformin is a widely used antidiabetic drug that has been recently shown to exert anti-calcific and anti-fibrotic effects in various tissues, in part by enhancing FOXO1 expression or activity [19–21; 25]. In contrast, AS1842856 is a selective small-molecule inhibitor of FOXO1 that disrupts FOXO1’s ability to repress downstream transcriptional targets lifting its inhibitory effect on pro-osteogenic RUNX2 [22].

**Fig. 4:**
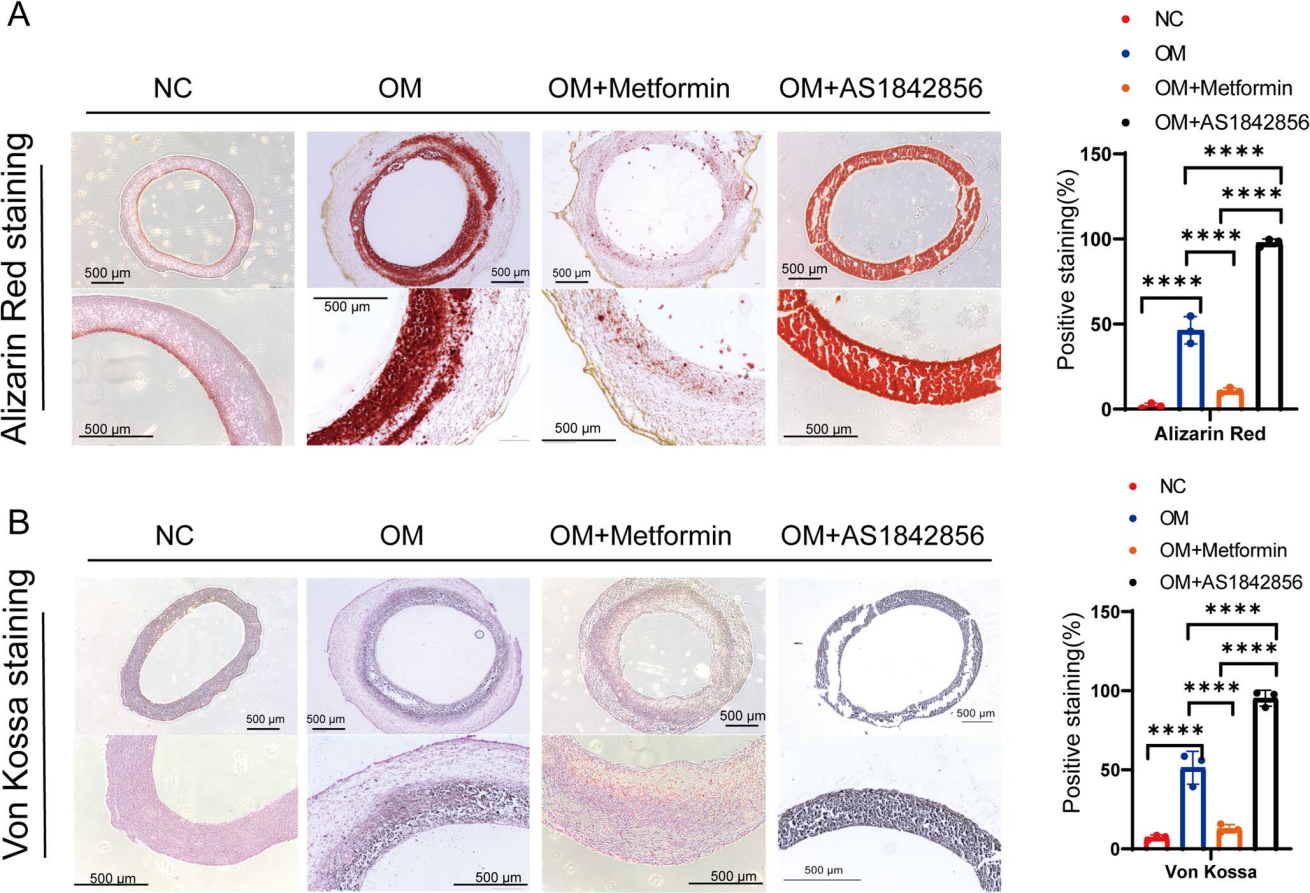
Histological staining and calcification 3D hiVICs tissue construct treated with OM and pharmacological agents. (**A**) Alizarin red staining of tissue constructs with or without OM, metformin and AS1842856 treatments and their quantification. *n* = 3 biological replicates. (**B**) Von Kossa staining in NC-treated tissue constructs, as well as constructs treated with OM, OM supplemented metformin and OM supplemented with AS1842856. *n* = 3 biological replicates. Statistical analysis was performed using one-way ANOVA followed by Tukey’s post hoc test for all comparisons. Asterisks indicate statistical significance: *P* < 0.05 (*), *P* < 0.01 (**), *P* < 0.001 (***), and *P* < 0.0001 (****)

As expected, Alizarin Red staining revealed extensive calcium deposition throughout the OM-treated constructs (Fig. 4A). Treatment with metformin significantly reduced calcium accumulation, with Alizarin Red staining levels nearly indistinguishable from the control (NC) group. Conversely, AS1842856 markedly exacerbated mineralization, with pervasive Alizarin Red staining observed across the construct, exceeding that of OM alone. Von Kossa staining further supported these observations (Fig. 4B). While OM alone induced moderate phosphate-based mineralization, AS1842856-treated constructs showed widespread, dense staining, indicating severe calcific transformation of the tissue. Metformin treatment, on the other hand, effectively suppressed the calcification of the constructs. Together, these findings demonstrate that osteogenic induction in 3D culture robustly promotes calcification and, which can be either exacerbated by FOXO1 inhibition (AS1842856) or effectively suppressed by metformin. These results highlight the utility of our 3D hiVIC model as a platform for treatment with pharmacological modulators.

### Differential Expressions of RUNX2 and FOXO1 in 2D Versus 3D Cultures

**Fig. 5:**
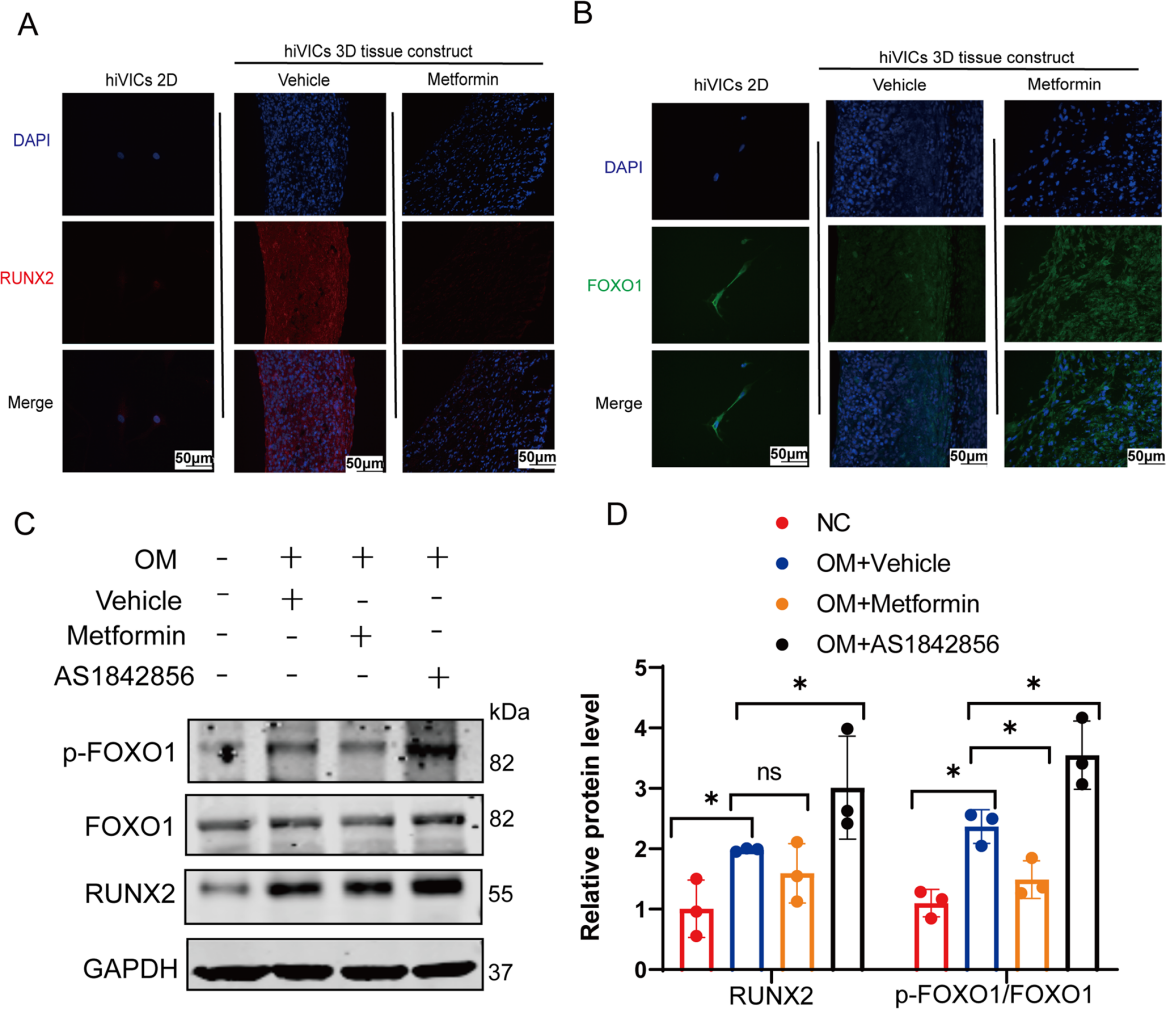
RUNX2 and FOXO1 levels in 2D and 3D cultures of hiVICs. (**A**) RUNX2 immunofluorescence in 2D culture, and 3D hiVICs constructs treated with OM and OM plus Metformin. (**B**) FOXO1 immunofluorescence in 2D culture, and 3D hiVICs constructs treated with OM and OM plus Metformin. (**C**) Western blot analyses for RUNX2 and p-FOXO1/FOXO1 on day 7 hiVIC tissue ring constructs with or without OM, metformin and AS1842856 treatments. *n* = 3 biological replicates. (**D**) Quantification of the western blots. Statistical analysis was performed using one-way ANOVA followed by Tukey’s post hoc test. Asterisks indicate statistical significance: *P* < 0.05 (*), *P* < 0.01 (**), *P* < 0.001 (***), and *P* < 0.0001 (****)

To investigate the differential calcification potential of hiVICs in 2D versus 3D cell culture environments, we examined the expression patterns of two key transcription factors involved in osteogenic differentiation—RUNX2 and FOXO1. Immunofluorescence staining revealed that in conventional 2D culture, hiVICs displayed weak nuclear localization of RUNX2 and high levels of FOXO1 expression (Fig. 5A–B), consistent with a non-calcifying, quiescent interstitial phenotype. However, when hiVICs were cultured in 3D self-assembled constructs and treated with osteogenic medium (OM), a marked increase in nuclear RUNX2 and a substantial reduction in FOXO1 staining were observed. These findings suggest that the 3D microenvironment not only promotes osteogenic gene expression but also represses anti-osteogenic signaling.

Furthermore, metformin supplementation—an agent known to inhibit valvular calcification [21] reversed the OM-induced calcification (Fig. 4). RUNX2 levels in metformin-treated 3D constructs was reduced compared to OM-only constructs, while FOXO1 levels were restored toward baseline (Fig. 5A–B), indicating that metformin modulates the RUNX2–FOXO1 axis to suppress calcification. These observations are consistent with the previous report that FOXO1 acts as a suppressor of osteogenesis, and its downregulation in 3D constructs facilitates RUNX2 activation and subsequent mineralization [22]. These results suggest that hiVICs exhibit distinct transcriptional regulation of osteogenic markers depending on the culture dimensionality by promoting RUNX2 expression and suppressing FOXO1 expression in 3D environments.

To further assess the early molecular response of 3D hiVIC constructs to osteogenic and pharmacological stimulation, we examined protein levels after 3 days of treatment, with OM and drugs added on day 4 and tissue rings collected on day 7. OM stimulation markedly increased the levels of RUNX2 and phosphorylated FOXO1 (p-FOXO1/FOXO1) compared with the NC group (Fig. 5C and D), indicating early activation of osteogenic signaling and FOXO1 inactivation. Metformin treatment significantly attenuated p-FOXO1 upregulation, whereas the FOXO1 inhibitor AS1842856 further enhanced RUNX2 and p-FOXO1 levels. These results demonstrate that short-term osteogenic stimulation dynamically regulates the FOXO1–RUNX2 axis and that pharmacological modulation of FOXO1 directly impacts osteogenic remodeling in 3D hiVIC constructs.

### Regulation of 3D hiVIC Construct Mechanical Properties by Calcification Modulators

**Fig. 6:**
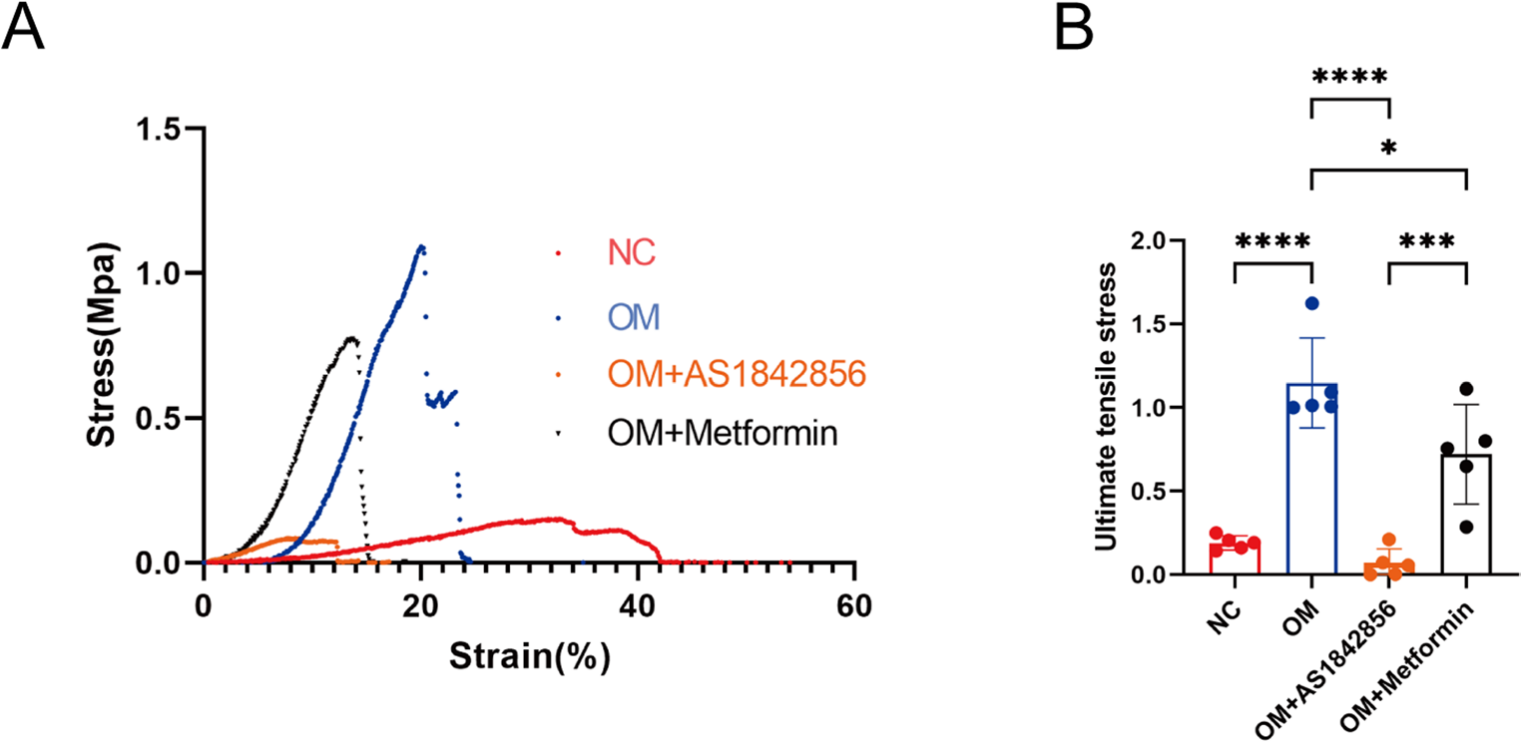
Mechanical testing of 3D hiVICs tissue construct with OM and drug treatment. (**A**) Representative stress-strain curves and maximum tensile stress values of tissue constructs with or without OM, metformin and AS1842856 treatments. (**B**) Quantification of mechanical test data. *n* = 5 biological replicates. Statistical analysis was performed using one-way ANOVA followed by Tukey’s post hoc test. Asterisks indicate statistical significance: *P* < 0.05 (*), *P* < 0.01 (**), *P* < 0.001 (***), and *P* < 0.0001 (****)

To evaluate the effects of osteogenic stimulation and pharmacological modulation on the biomechanical properties of engineered tissue ring constructs, we performed uniaxial tensile testing on 3D hiVIC tissue rings treated with normal control (NC), osteogenic medium (OM), OM plus metformin, or OM plus AS1842856. Representative stress–strain curves showed that OM-treated constructs exhibited increased mechanical integrity compared to controls (Fig. 6A), reflecting tissue stiffening associated with calcification and ECM remodeling.

Quantitative assessment of ultimate tensile stress demonstrated a marked increase in OM-treated constructs compared with NC controls (Fig. 6B). Treatment with metformin reduced tensile strength relative to OM alone, but values remained above NC, suggesting that metformin mitigates calcification while potentially influencing extracellular matrix (ECM) maturation or collagen crosslinking. In contrast, AS1842856 treatment led to a pronounced loss of tensile strength and overall mechanical integrity, consistent with severe structural disruption. The enhanced tensile performance observed in OM-treated constructs may result from concurrent ECM deposition and collagen fibril crosslinking during early mineralization, which transiently reinforce the matrix. In comparison, both calcification-promoting and calcification-attenuating interventions appear to disrupt this balance—either by inducing excessive mineral accumulation that increases brittleness (AS1842856) or by limiting ECM crosslinking during matrix maturation (metformin)—thereby reducing tensile performance relative to the OM peak.

## Discussion

Most existing 3D aortic valve models are designed for prosthetic valve development rather than recapitulating the pathological progression of CAVD, and typically rely on synthetic scaffolds.[12,13] Here we developed a self-assembled 3D tissue construct model using hiVICs in this study. This model enables direct assessment of ECM remodeling and calcification and provides a simple platform for mechanistic studies and drug screening. We also investigated the mechanisms underlying the significant differences in calcification rate between hiVICs cultured in 2D versus 3D environments.

Our molecular characterization revealed dynamic regulation of the FOXO1–RUNX2 axis in response to dimensional context and pharmacological manipulation. Compared to primary VICs (pVICs), hiVICs cultured in 2D exhibited lower RUNX2 and higher FOXO1 levels, potentially explaining their inability to undergo calcification under OM stimulation. However, when hiVICs were cultured in 3D tissue constructs, we observed a marked increase in RUNX2 and concomitant decrease in FOXO1 expression, which correlated with the onset of mineralization under osteogenic medium. The present findings validate the previously reported AKT–FOXO1–SMURF2–RUNX2 regulatory axis in pVICs and further extend its relevance to two and three-dimensional hiVICs tissue-ring cultures, suggesting that this pathway remains functionally conserved under 3D conditions and contributes to osteogenic activation of hiVICs. Notably, under two-dimensional conditions, SMURF2 expression in hiVICs was higher than in pVICs, consistent with its role in promoting RUNX2 ubiquitination and degradation, potentially limiting the osteogenic capacity of 2D hiVICs. Given that SMURF2 was not detected in 3D constructs, the upregulation of RUNX2 in 3D culture suggests that dimensional changes may attenuate SMURF2’s inhibition of RUNX2. Overall, the 3D microenvironment likely enhances cell–cell and cell–matrix interactions, activating PI3K/AKT signaling and relieving FOXO1-mediated repression of RUNX2 [26]. Pharmacological perturbations further support this regulatory relationship: treatment with FOXO1 regulator metformin [25] significantly attenuated calcification, nearly restoring the baseline phenotype, accompanied by increased FOXO1 expression. Conversely, treatment with the selective FOXO1 inhibitor AS1842856 exacerbated mineralization and led to severe tissue collapse, reinforcing FOXO1’s protective role in maintaining matrix homeostasis and preventing pathological mineral deposition. Metformin shows promising translational potential in CAVD. These findings provide mechanistic rationale for repurposing metformin as a therapeutic candidate for CAVD.

Mechanical testing revealed that moderate calcification was associated with enhanced tensile strength, whereas advanced mineral accumulation led to reduced mechanical integrity. These results highlight the complex interplay between biochemical signaling, ECM remodeling, and mechanical properties in 3D hiVICs construct. While osteogenic conditions enhance construct stiffness through mineral and matrix accumulation, perturbation of the calcification pathway—either through inhibition (metformin) or deregulated activation (AS1842856)—appears to compromise mechanical maturation. This highlights the importance of incorporating mechanical metrics in addition to histological and molecular endpoints when evaluating therapeutic agents for CAVD. In previous studies, drug screening efforts for calcific aortic valve disease have predominantly focused on identifying compounds that directly inhibit calcium deposition or osteogenic differentiation of valvular interstitial cell [27].However, this approach may overlook a category of therapeutics that do not necessarily block mineralization itself but can modulate the biomechanical properties of the valve tissue, particularly by attenuating the pathological increase in stiffness associated with calcification progression Such agents, including antifibrotic drugs or Angiotensin receptor blockers, may still benefit valve function by preserving tissue compliance and delaying functional deterioration [28,29]. Notably, the 3D hiVIC-based model presented in this study enables quantitative assessment of tissue elasticity through uniaxial tensile testing, thus offering a novel platform for screening drugs based on their impact on mechanical performance. This expanded capability broadens the scope of therapeutic discovery in CAVD and supports the development of more nuanced treatment strategies that may slow disease progression even in the absence of complete calcification inhibition.

In summary, the intermediate phenotype of hiVICs may limit their osteogenic potential compared with primary VICs, likely due to incomplete molecular and epigenetic maturation in 2D culture. However, the 3D self-assembled environment enhances cell–cell and cell–matrix interactions, promoting RUNX2 activation and mineralization. Further optimization of differentiation protocols or co-culture with valve endothelial cells could improve hiVIC maturity and model fidelity. Given that hiVICs can be derived from patient-specific iPSCs, our 3D construct model allows personalized drug screening. hiPSC-derived cells retain the genetic characteristics of the donor, thereby potentially reflecting variations in calcification propensity and drug responsiveness among different patients. Consequently, this platform is not only suitable for evaluating the effects of anti-calcification drugs under controlled conditions but also holds potential for advancing treatment strategies for CAVD by identifying optimal patient-specific therapeutic modalities.

### Limitations of the Study

This study has limitations. HiVICs generated through the directed differentiation protocol exhibit an intermediate phenotype when compared to primary VIC and are less prone to calcification in monolayer cultures. This suggests that the differentiated hiVICs may not fully recapitulate the functional maturity of adult valvular interstitial cells, possibly lacking essential transcriptional programs or epigenetic cues required for osteogenic responsiveness in simplified environments. Optimizing differentiation protocols to enhance VIC maturity, or co-culturing with other valve-resident cell types such as valve endothelial cells (VECs), may help overcome this limitation. While our 3D model captures key aspects of calcification in vitro, it remains unclear whether the constructs possess the capacity to undergo mineralization in vivo under physiological conditions. Future studies should explore the potential of hiVIC-derived implantable constructs to calcify in vivo [30], thereby enabling the establishment of a patient-specific CAVD model.

## Supplementary Information

Below is the link to the electronic supplementary material.

Supplementary Material 1 (PDF 1.12 MB)

Supplementary Table 1 (DOCX 15.7 KB)

## Data Availability

The data from the study are available from the corresponding authors upon reasonable request.
