# Supplementary figures and images for: Construction of a Three-Dimensional Calcific Aortic Valve Disease Model Using Human iPSC-Derived Valvular Interstitial Cells
Source: Stem Cell Rev Rep. 2025 Dec 4;22(2):963–75. doi: 10.1007/s12015-025-11030-3 (PMC12858608; doi:10.1007/s12015-025-11030-3)

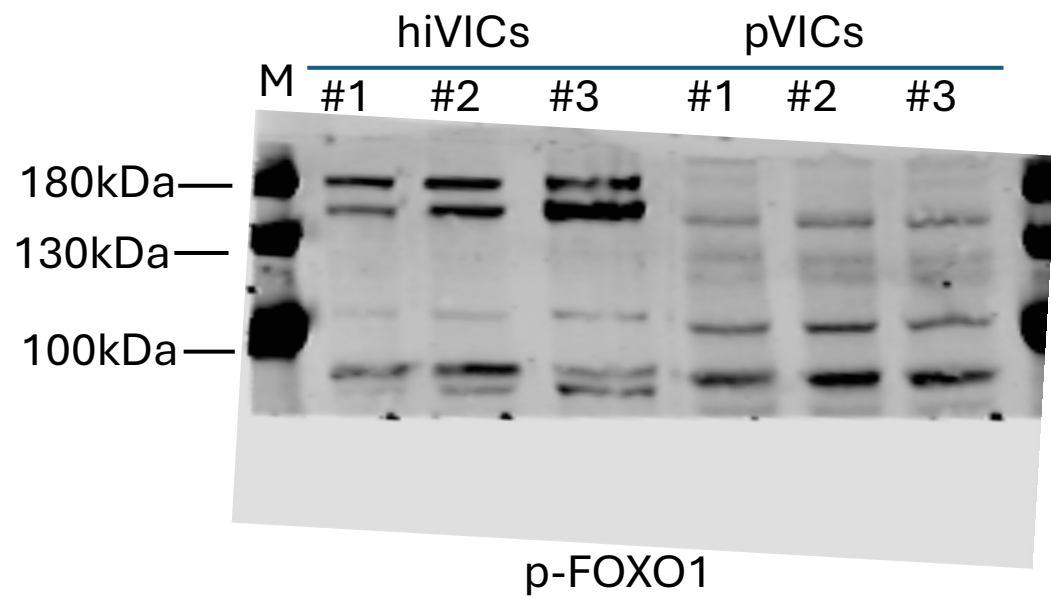

Figure 2D

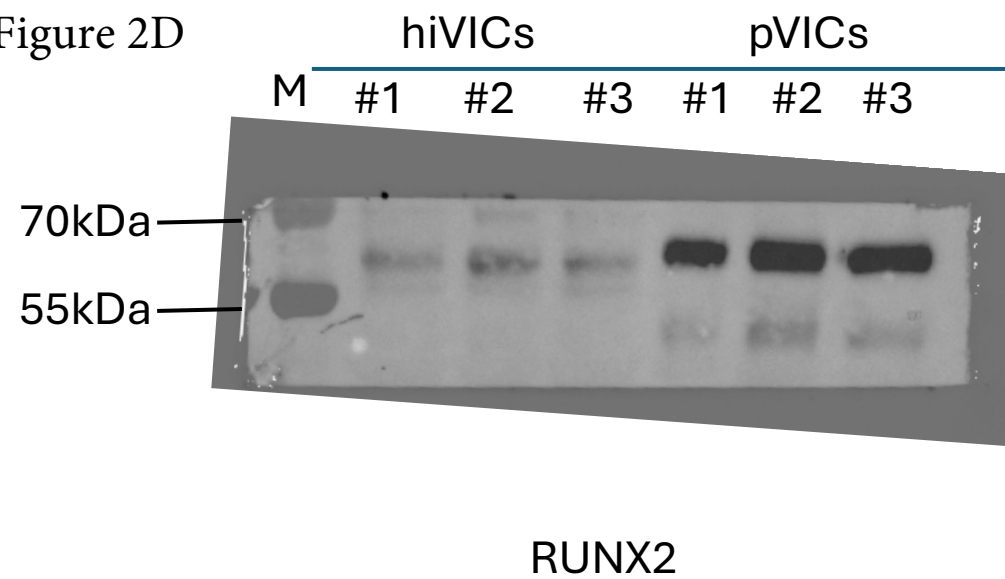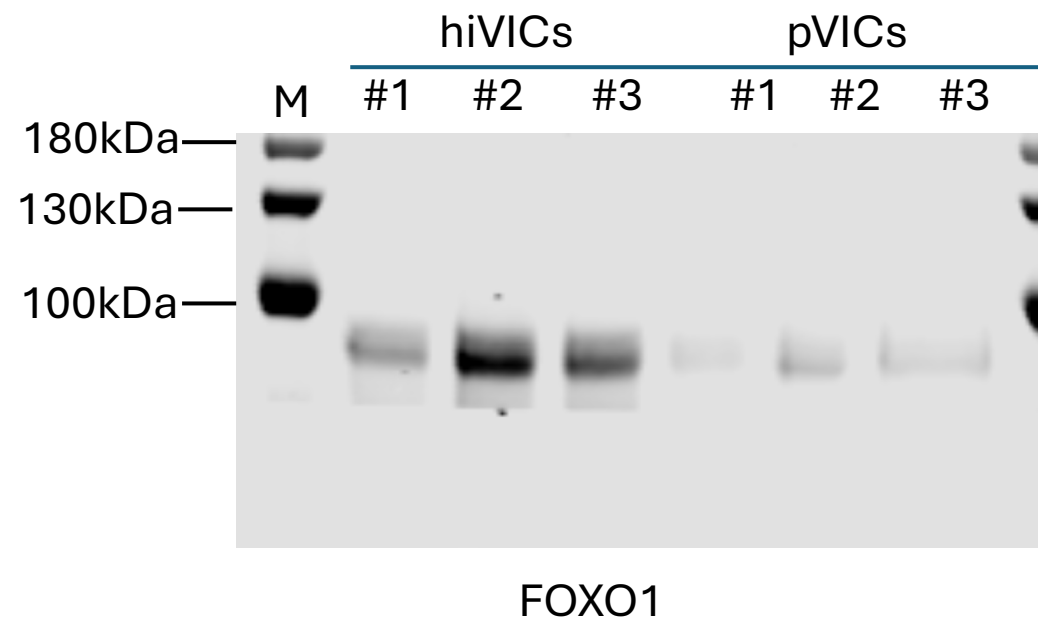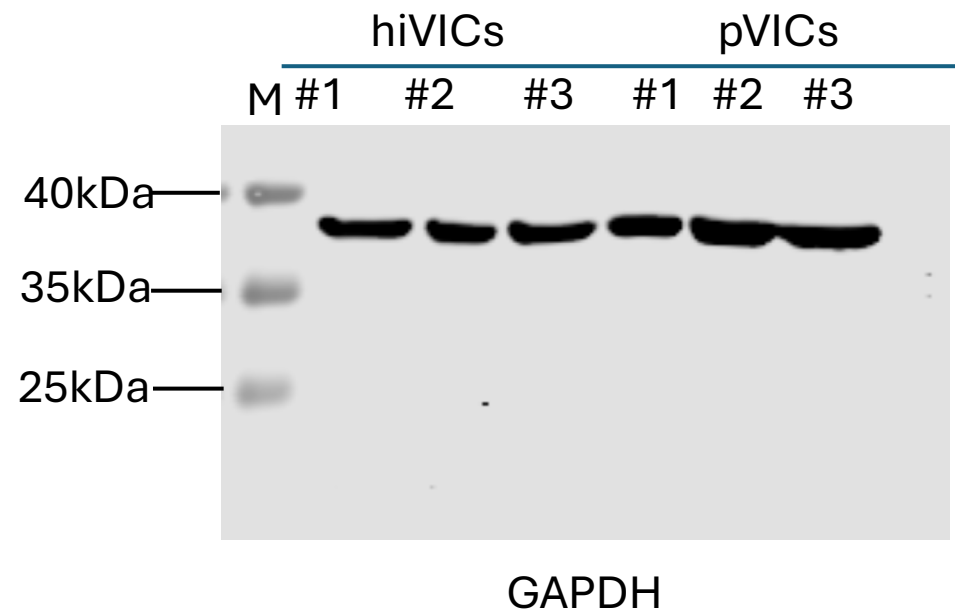

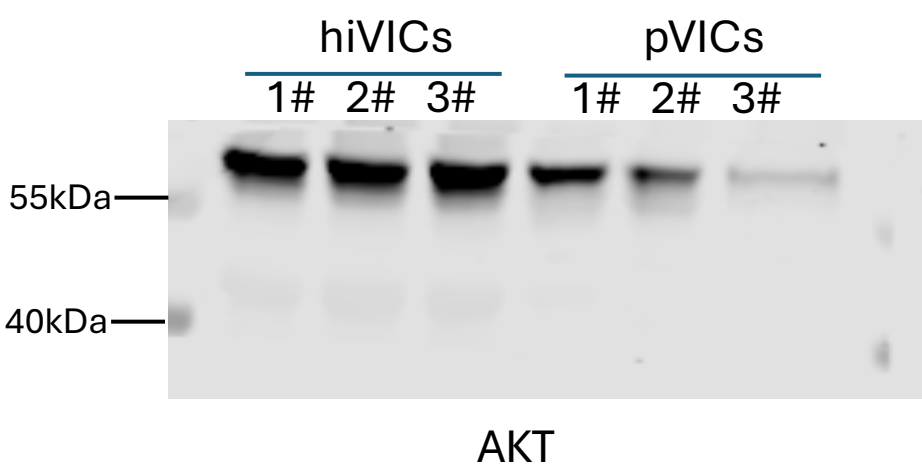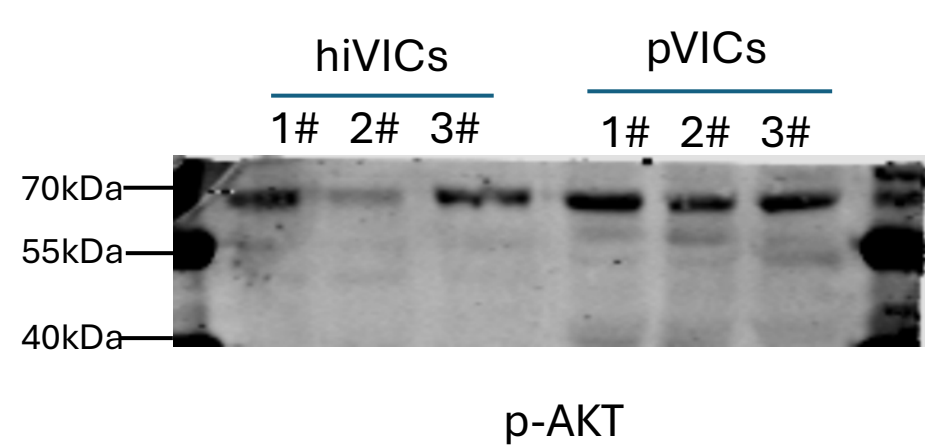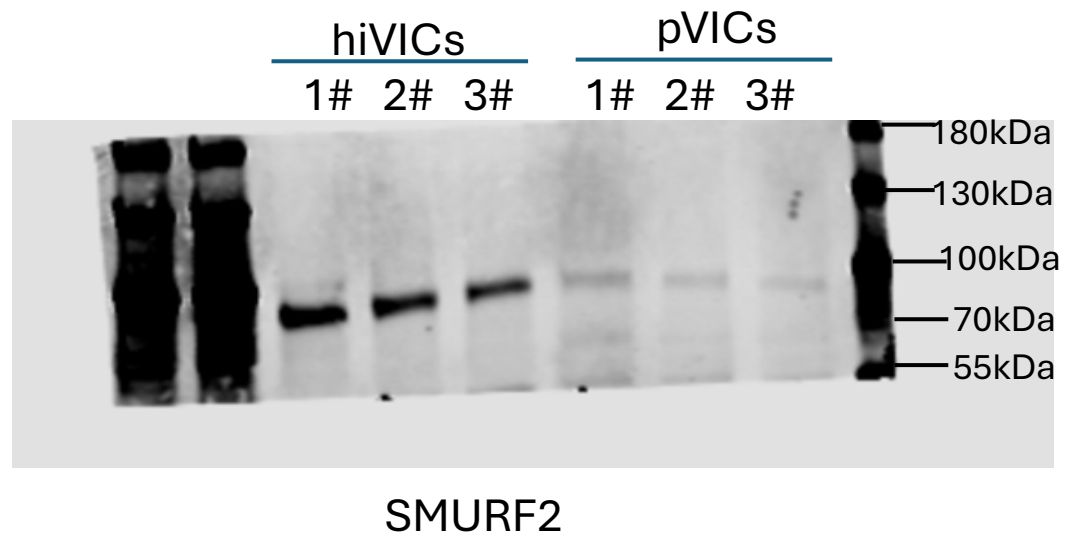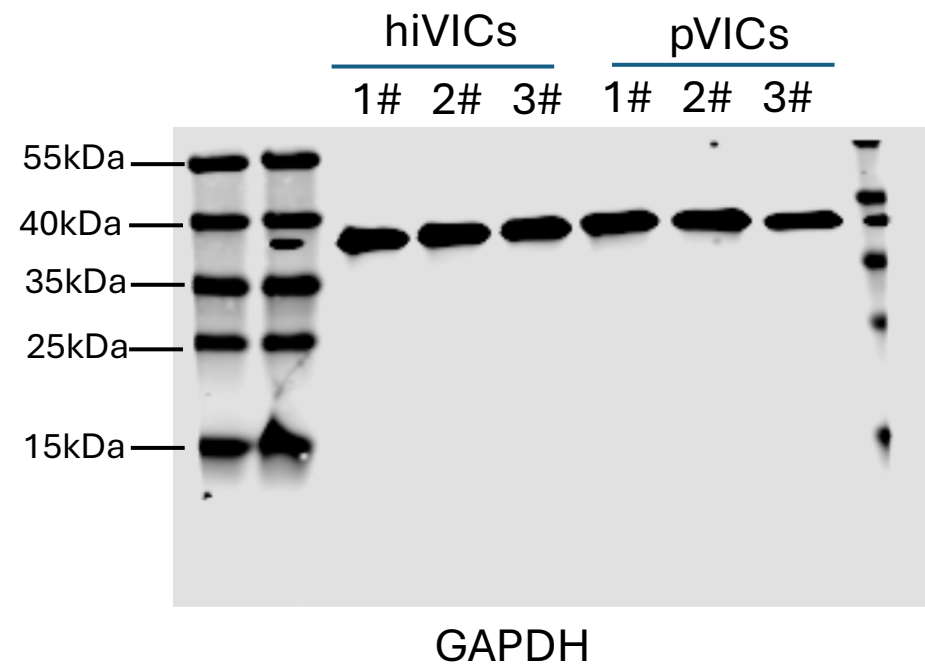

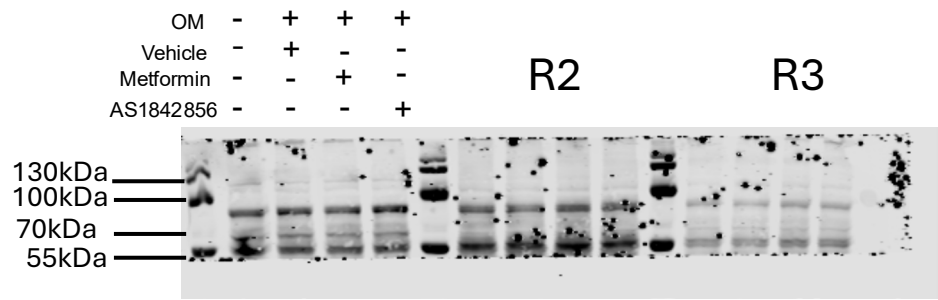

FOXO1

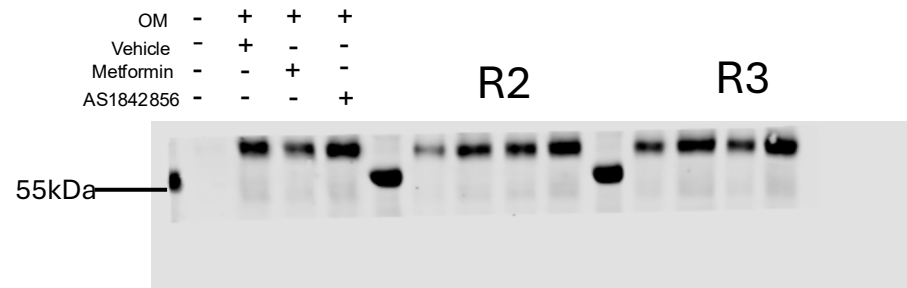

RUNX2

Figure 5C

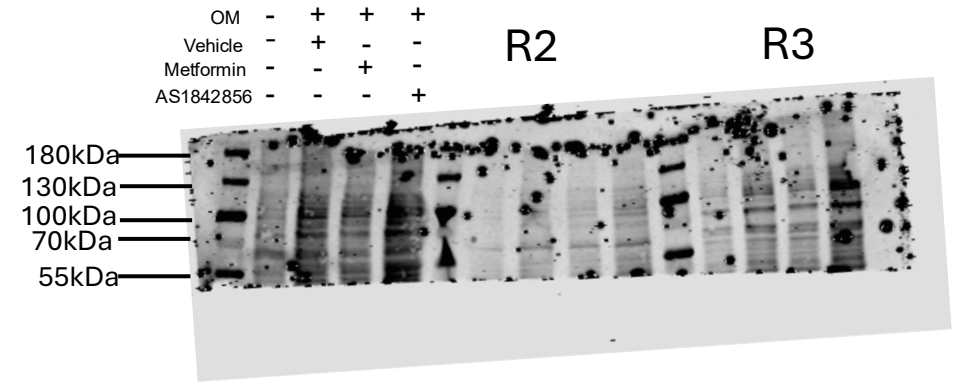

p-FOXO1

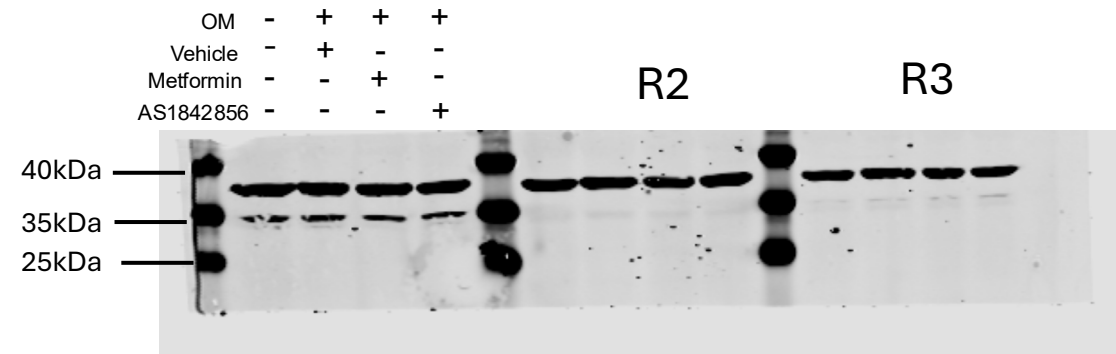

GAPDH

Supplement: Supplementary file 1 — Supplementary Material 1 (PDF 1.12 MB) [file 12015_2025_11030_MOESM1_ESM.pdf]
