# Supplementary material for: Construction of a Three-Dimensional Calcific Aortic Valve Disease Model Using Human iPSC-Derived Valvular Interstitial Cells
Source: Stem Cell Rev Rep. 2025 Dec 4;22(2):963–75. doi: 10.1007/s12015-025-11030-3 (PMC12858608; doi:10.1007/s12015-025-11030-3)
Supplement: Supplementary file 2 — Supplementary Table 1 (DOCX 15.7 KB) [file 12015_2025_11030_MOESM2_ESM.docx]

**Supplementary Table 1**

| **Primary name** | **Forward 5′‐3′** | **Reverse 5′‐3′** |
| --- | --- | --- |
| ***h-GAPDH*** | **TGTTGCCATCAATGACCCCTT** | **CTCCACGACGTACTCAGCG** |
| ***h-OCT-4*** | **AGCGACTATGCACAACGA** | **CCAGAGTGGTGACGGAGA** |
| ***h-SOX-2*** | **CAGGAGTTGTCAAGGCAGAG** | **CCGCCGATGATTGTTATT** |
| ***h-αSMA*** | **CTATGAGGGCTATGCCTTGCC** | **GCTCAGCCAGTAGTAACGAAGGA** |
| ***h-VIMENTIN*** | **GCAAAGATTCCACTTTGCGT** | **GAAATTGCAGGAGGAGATGC** |
| ***h-RUNX2*** | **GCCTTCCACTCTCAGTAAGAAG** | **CCTGGGGTCTGAAAAAGGG** |
| ***h-FOXO1*** | **TCGTCATAATCTGTCCCTACACA** | **CGGCTTCGGCTCTTAGCAAA** |
| ***h-TAGLN*** | **TCCAGGTCTGGCTGAAGAATGG** | **CTGCTCCATCTGCTTGAAGACC** |
| ***h-COL3A1*** | **TGGTCTGCAAGGAATGCCTGGA** | **TCTTTCCCTGGGACACCATCAG** |
